# The effectiveness of luteal phase support with cyclogest in ovarian stimulated intra uterine insemination cycles: A randomized controlled trial 

**Published:** 2013-04

**Authors:** Bibi Shahnaz Aali, Sakineh Ebrahimipour, Siavash Medhdizadeh

**Affiliations:** 1 *Physiology Research Center, Department of Obstetrics and Gynecology, Afzalipour Hospital, Kerman University of Medical Sciences, Kerman, Iran.*; 2 *Department of Obstetrics and Gynecology, Afzalipour Hospital, Kerman University of Medical Sciences, Kerman, Iran.*; 3 *Dentistry School, Kerman University of Medical Sciences, Kerman, Iran.*

**Keywords:** *Luteal phase*, *Progesterone*, *Intrauterine insemination Infertility*

## Abstract

**Background:** Controlled ovarian stimulation combined with intra uterine insemination (IUI) is a convenient treatment of infertility with a success rate of 11%. The clinical observation and pattern of progesterone secretion in this method is suggestive of luteal phase defect and postulated as an implicating factor of treatment failure.

**Objective:** To investigate the efficacy of luteal phase support with intravaginal cyclogest in women undergoing controlled ovarian stimulation combined with intrauterine insemination.

**Materials and Methods:** In this single-blinded clinical trial, 196 consecutively seen women eligible for the study protocol, were randomized to receive either intravaginal progesterone (cyclogest pessary, Actavis) or no medication in luteal phase. Blood samples were collected and serum progesterone level in 7th and 11th day of the cycle, biochemical and clinical pregnancy and luteal phase duration were compared in case and control groups.

**Results:** The mean age in case and control group was 28 and 27.9 years, respectively and the most frequent cause of infertility was unexplained. Additionally, ovulatory dysfunction was the most common cause of female infertility in both groups. Based on these variables, there was no statistically significant difference between the two groups. Mean serum progesterone level in the case group were 48.34 and 34.24nmol/day on day 7 and 11 after insemination, respectively and both values were significantly higher than the control group. There was no difference between the two groups in terms of biochemical and clinical pregnancy. Luteal phase duration in the case group was significantly longer than the control group.

**Conclusion:** Luteal phase support by Cyclogest pessary increases progesterone level and prolongs the luteal phase, but does not affect success rate of IUI cycles in terms of achieving pregnancy.

## Introduction

Based on the studies conducted, 10-15% of couples are infertile worldwide. Given the psychological and financial burden of this problem on the couples and their relatives, several treatment modalities have been considered for resolving it. Currently, about 3 million couples suffer from infertility in Iran and about 15% is added to their population annually. Iran is now among the world’s top ten countries in infertility treatment and more than 90% of infertile couples can have children with various methods of treatment. Some studies show that most infertile couples become disappointed to continue the treatment due to psychological conditions and financial issues arising from failure of treatment (1, 2). Infertility may occur as a primary or secondary problem and its main reasons are: ovulatory dysfunction (15%), tubal, uterine and peritoneal factors (30-40%), male factor (30-40%) and unexplained in 5-10% of cases (3, 4). Currently used modalities to treat infertility are: controlled ovarian stimulation with or without IUI, surgery and assisted reproduction techniques (4).

Intrauterine insemination (IUI) is one of the most common and effective infertility treatments that its utilization has increased in the recent decades in regard to the convenience and low rate of complications (5, 6). Ovulation is usually induced by clomiphene citrate either with or without gonadotropins. Letrozole has been shown to be a suitable alternative for clomiphene citrate (7). 

Chance of getting pregnant in IUI cycle is about 11%. HCG is injected when the diameter of dominant follicle is 17-20mm and insemination is performed 36 hours after hCG injection (3, 4). In normal ovulatory cycles, 2 days after LH surge, progesterone level increases gradually and reaches its maximum level of about 75 nmol/lit on 8^th^ day of LH surge, then starts to decline from day 9 reaching to about half level (40 nmol/lit) on the 10^th^ day. Finally it returns to its baseline level 5 days later. This pattern is different in controlled ovarian stimulation cycles, so that progesterone level maximizes 6-8 days after hCG injection and then suddenly drops. If pregnancy does not occur progesterone reaches its baseline level in about 4 days later. Thus, luteal phase duration is 1-3 days shorter than the simultaneous ovulatory cycles.

Also, in clinical practice it is usual to see menstruation occurs sooner in non-conceiving IUI cycles in comparison to normal ovulatory cycles. It is postulated that the pattern of progesterone secretion in IUI cycles can be representative of luteal phase defect and a reason for IUI failure (8). Therefore, administration of progesterone in luteal phase has been regarded as a modality to abate this obstacle. However, optimal progesterone level in ovulation induction cycles is still not clear (9). Increase in the number of progesterone receptors in uterotubal junction of sows after intrauterine insemination has been considered as a contributing factor in sperm transportation and the fertilization process and hence achievement of pregnancy (10).

All these evidences challenge the administration of progesterone after IUI in stimulated ovarian cycles to increase their success rate. In fact, some researchers have shown a higher pregnancy rate and a lower frequency of abortion with progesterone administration in luteal phase of controlled ovarian stimulation cycles (4). Cohlen in an Evidence Based Review recommended supporting the luteal phase in IUI cycles (6). Besides, in a clinical trial conducted by Erdem *et al* on 214 patients, it was found that progesterone gel can increase success rate of IUI cycles (11). 

Moreover, Montville *et al* study on 121 women suffering from polycystic ovary syndrome and infertility, indicated effectiveness of luteal phase support by progesterone (12). On the other hand, a study conducted by Ebrahimi *et al* on 200 infertile couples with unexplained infertility treated with IUI, indicated that cyclogest administration in luteal phase has no effect on the success rate (13). Different route of administration and variable formulation of progesterone and also the biological composition of women receiving the medication can affect the findings. Therefore we aimed to study the effectiveness of intravaginal progesterone, cyclogest pessary, in stimulated ovarian IUI cycles. 

## Materials and methods

In a single blinded clinical trial, of 300 women candidate for IUI attending Afzalipour IVF center, Kerman, Iran from April 2010 to December 2011, 200 eligible cases entered the study. Women with laparoscopic or radiologic evidences of patent tubes, age lower than 40 years, FSH<12 Iu/L and duration of infertility less than 5 years, less than two previous failed attempt of IUI and no history of chronic liver, kidney and heart disease were included. 

Besides, Just couples with a sperm count of >10 million/mL entered the study, considering the important role of this factor in achievement of pregnancy in IUI cycles (14). The study protocol was approved by the ethical committee of Kerman Medical University (registration number: K/88/108). Informed consent was taken from each patient and block randomization was utilized with a block size of 4 for minimizing the effect of confounding factors. Baseline transvaginal ultrasonography was performed for all patients on day 3 of the menstrual cycle and treatment started based on patient’s characteristics and record of her previous cycle, with either a single intramuscular injection of 75-150 IU highly purified HMG (Merional, IBSA) per day or clomiphene citrate 100 mg daily for 5 days followed by single injection of Merional on day 8 and 9 of the cycle. Ovarian response was assessed with vaginal sonography from day 8 of each cycle, by two expert IVF specialists. 

When at least one follicle reached 18 mm in diameter, ovulation was triggered with intramuscular injection of 10,000 IU hCG (Pregnyl; Organon) and 34-36 hours later IUI was done. Cycle was cancelled if no follicle or more than 4 follicles larger than 17 mm was observed. 196 women met the criteria of the study protocol and randomized to treatment (Cyclogest) or no treatment groups based on the order of the letters in each blocks (A and B) by a nurse who was responsible for dispensation and instruction of the medication. The researcher was blind about the individuals who received the medication. 

Case group consisted of 99 women received cyclogest pessary (400 mg, Actavis) per vagina daily for ten days, starting from the day after IUI, while 97 patients in control group received no additional medication. 2 mL blood sample was taken from each patient on days 7 and 11 after HCG injection. 6 and 7 patients of case and control group, respectievely did not attend for blood collection on day 7 and five patients in either group refused to give blood on day 11. Blood samples were allowed to clot at room temperature and then centrifuged. 

Serum specimens were frozen at -20^o^C until the completion of sample size. Serum progesterone was measured by electrochemiluminescent method. In order to evaluate the reliability of laboratory reports, one blood sample per 10 patients was examined blindly for the second time and also 10 blood samples were evaluated for the assessment of hormone in the reference laboratory of Kerman University. 

**Figure 1:**
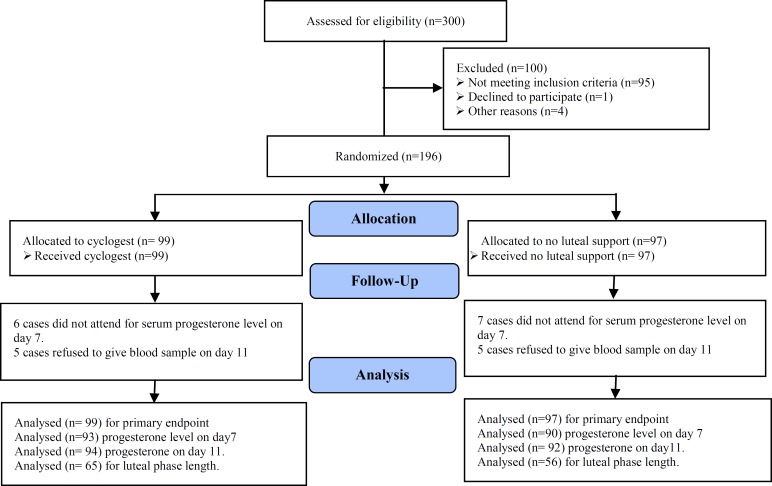
Consort flow diagram of RCT

Correlation coefficient of 98% for the former and 95% for the latter were considered as the reliability of laboratory performance. The number of biochemical and clinical pregnancy was compared in the groups as the major endpoints. Biocchemical pregnancy was considered as positive β-hCG test on day 14 or 17 after IUI and clinical pregnancy regarded as the observation of gestational sac on ultrasound scan done 21-30 days after IUI. Also, duration of luteal phase and progesterone level were compared as the secondary or minor endpoints. Luteal phase was calculated from the day of IUI till the occurrence of menstruation in unsuccessful cycles (Figure 1).

**Statistical analysis**

SPSS 17 was used for data analysis. Variables were described first and then compared in the groups utilizing independent t-test or its nonparametric equivalent, as required and chi test. P<0.05 was considered as significant.

## Results

Of 196 patients studied, 99 and 97 belonged to case and control group, respectively. The mean age was 28 years in the case and 27.9 years in the control group. Also, the mean age of husband was 31.9 years in case group versus 31.8 in the control. Patients were divided into 4 categories based on the cause of infertility. Comparison of the two groups regarding infertility cause and mean age is presented in table I. 

As it is evident in the table, mentioned variables did not differ significantly in the groups. Most frequent cause of female infertility in both groups was ovulatory dysfunction, i.e., 32 patients in control group (76.2%) and 30 patients in case group (77%). Mean blood progesterone levels on 7^th^ and 11^th^ day in the two groups are presented in figure 1. As it is shown, progesterone level in the case group was significantly higher than the control group. 

In 14.4% of the control and 16.2% of the case group, β-hCG test became positive. However, only 9 patients (9.3%) in the control and 12 patients (12.1%) in the case group achieved clinical pregnancy approved by ultrasound (Table II). Although, pregnancy happened in a higher number of women who received progesterone in comparison to the control group, this difference was not statistically significant.

Most biochemical pregnancies occurred in couples with unexplained infertility (56.7%), while 33.3% were among patients with female factor (10 cases) and just 10% (3 cases) in those with male factor infertility. Similarly, clinical pregnancy was observed in a higher number of patients with unexplained infertility and none of couples with mixed male and female factor achieved pregnancy (Table III). In unsuccessful cycles, duration of luteal phase was calculated from the day of IUI to the day of menstruation in 56 patients of control and 65 patients of case group, who had already regular menstrual cycles. The mean duration of luteal phase was 14.46±1.7 days in case and 13.05±1.4 days in control group (p<0.037).

**Table I:** Demographic data of women undergoing COH+IUI for infertility

** Group**			**With Cyclogest**	**Without Cyclogest**	**p-value**
**Variable**					
Age (years)			28	27.9	0.85
Duration of infertility (years)			3.97	4.07	0.81
Type of infertility					0.79
		Primary	88 (89.9)	86 (88.7)	
		Secondary	10 (10.1)	11 (11.3)	
Infertility diagnosis					0.91
	Female factor		36 (36.4)	38 (39.2)	
	Male factor		16 (16.2)	16 (16.5)	
	Mixed male and female factor		3 (3)	4 (4.1)	
	Unexplained		44 (44.4)	39 (40.2)	
Baseline FSH (Iu/L)			6.9 ± 2.4	7.2 ± 2.1	0.65
Baseline LH (Iu/L)			5.5 ± 4.5	6.123 ± 3.8	0.67
Baseline E2 (pg/ml)			51.9 ± 45	50.8 ± 38	0.74

(Independent t-test, λ^2^ test applied

**Table II:** Comparison of achieving pregnancy in the groups

** Group**	**With Cyclogest** **N (%)**	**Without Cyclogest** **N (%)**	**p-value**
**Variable**			
Chemical pregnancy	16 (16.2)	14 (14.4)	0.73
Clinical pregnancy	12 (12.1)	9 (9.3)	0.52

(λ^2^ test applied

**Table III:** Achievement of pregnancy in subgroups of infertility

** Infertility factor**	**Unexplained** **N (%)**	**Female** **N (%)**	**Male** **N (%)**	**Mixed male and female** **N (%)**	**Total** **N (%)**	**p-value**
**Pregnancy**						
Biochemical	17 (56.7)	10 (33.3)	3 (10)	0 (0)	30 (100)	0.33
Clinical	14 (66.7)	5 (23.8)	2 (0)	0 (9.5)	21 (100)	0.16

(λ^2^ test applied

**Figure 2:**
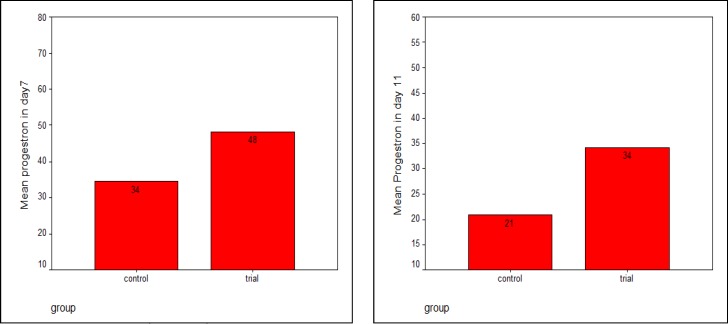
Progesterone level on 7^th^ and 11^th^ day of IUI in the groups (nmol/l).

## Discussion

In terms of bichemical and clinical pregnancy we found no significant difference in case and control group. In contrast, in a study conducted in Turkey, luteal phase support clearly increased clinical pregnancy rate (11). However, that study just recruited women with unexplained infertility, while in our study four causes of infertility, i.e., female factor, male factor, mixed and unexplained were included. However, our results was in line to a study conducted in Iran on women with unexplained infertility which showed no significant difference in pregnancy rate in cases with luteal phase support (13). 

Also in the study conducted by Montvillein and Khabbaz in 2010 on infertile women who received luteal phase support during IUI cycles, no significant difference in pregnancy rate was observed (12). Also, Kyrou *et al* in a study on 400 IUI cycles found no significant difference in cases with and without luteal support, with micronized progestrone although in that study induction of ovulation was done just by clomiphene citrate (15). The discrepancy in the findings of the studies can be attributed to the differences between the study population, protocol of ovulation induction and also progesterone product used for luteal support.

Of total pregnancies achieved in our study, most occurred in the subgroup with unexplained infertility. This finding is partly related to the greater population of them in our study and partly reflects the absence of an underlying cause which can prevent pregnancy. Mean blood progesterone level on 7^th^ day of IUI was 34.49 and 48.34 in control and case group, respectively and the difference was statistically significant (p=0.000). 

Likewise, on the day 11 after IUI blood progesterone level in patients with luteal phase support was significantly higher than the control group. A study conducted by Costello et al in 2004, revealed a higher blood progesterone levels pursuant to luteal support, which was similar to our study (16). Further our study showed a longer luteal phase while using cyclogest. Although there is no evidence of its beneficial effect at the present time, it might stabilize the endometrium in the absence of pregnancy and make it ready for upcoming cycles. We conclude that although administration of cyclogest can increase the progesterone level and consequently the duration of lutal phase in IUI cycles but this outcome does not affect the pregnancy rate in these cycles. 
